# 

**Published:** 1883-07

**Authors:** 


					﻿ESTABLISHED IN 1B55,
4	JULY. «83.	vSTS’™
____ATLANTA
MEDICAL’ REGISTER.
(ATLANTA MEDICAL AND S CRGICAL JO VENAL.)
ZZITEZ B'S"
J. H. LOGAN, A.M., M.D.
H. H. DICKSON, PUBLISHER. TERMS, $2.50 A YEAR IN ADVANCE.
ATLANTA, GEORGIA:
DICKSON, BOOK ABD JOB IB1NTEK AND BOOKBINDER.
1883.
				

